# Antibodies against nephronectin ameliorate anti‐type II collagen‐induced arthritis in mice

**DOI:** 10.1002/2211-5463.12758

**Published:** 2019-11-24

**Authors:** Shigeyuki Kon, Machiko Honda, Kiyoshi Ishikawa, Masahiro Maeda, Tatsuya Segawa

**Affiliations:** ^1^ Department of Molecular Immunology Faculty of Pharmaceutical Sciences Fukuyama University Japan; ^2^ Immuno‐Biological Laboratories Fujioka Japan

**Keywords:** antibody, anti‐type II collagen‐induced arthritis, autoimmune diseases, ELISA, nephronectin

## Abstract

The extracellular matrix protein nephronectin (Npnt) is known to be critical for kidney development, but its function in inflammatory diseases is unknown. Here, we developed a new enzyme‐linked immunosorbent assay system to detect Npnt in various autoimmune diseases, which revealed that plasma Npnt levels are increased in various mouse autoimmune models. We also report that antibodies against the α8β1 integrin‐binding region of Npnt protect mice from anti‐type II collagen‐induced arthritis, suggesting that Npnt may be a potential therapeutic target molecule for the prevention of autoimmune arthritis.

## Graphical abstract

The role of extracellular matrix protein nephronectin (Npnt) in inflammatory diseases is unknown. In this study, we show that plasma Npnt levels are increased in various mouse autoimmune models and antibodies against the α8β1 integrin‐binding region attenuated the development of anti‐type II collagen‐induced arthritis in mice.

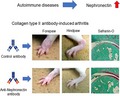

**Abbreviations**
CAIA: collagen antibody‐induced arthritis
ConA: concanavalin A
EAE: experimental autoimmune encephalomyelitis
ECM: extracellular matrix
EGF: epidermal growth factor
ELISA: enzyme‐linked immunosorbent assay
HE: hematoxylin and eosin
LPS: lipopolysaccharide
MAM: meprin/A5‐protein/PTPmu
MOG: myelin oligodendrocyte glycoprotein
Npnt: nephronectin
Npnt‐FD: nephronectin‐functional domain
OPN: osteopontin
PLP: proteolipid protein
RA: rheumatoid arthritis
SUMO: small ubiquitin‐related modifier
TLR: Toll‐like receptor

The extracellular matrix (ECM) primarily functions to support the intercellular space; however, recent studies suggest that the ECM regulates various cellular functions including cell differentiation, cell growth, survival, adhesion, and migration by mediating integrin receptors 1, 2, 3. A wide variety of ECM proteins have the same primary sequence motif, a tripeptide, Arg‐Gly‐Asp (RGD) motif for integrin binding 4. The expression of some ECM components is increased during autoimmune diseases 5, 6. For example, we previously reported that osteopontin (OPN), an ECM protein containing an RGD motif, is highly expressed in inflammatory diseases and critically involved in autoimmune diseases, including collagen antibody‐induced arthritis (CAIA) 7, 8 and concanavalin A (ConA)‐induced hepatitis 9. Therefore, aberrant ECM expression facilitates disease development.

An ECM protein, nephronectin (Npnt), is expressed in kidneys and hair follicles 10, 11. Npnt consists of five epidermal growth factor (EGF)‐like repeats in N‐terminal region, a linker segment containing an RGD integrin‐binding site, and a meprin/A5‐protein/PTPmu (MAM) domain in C‐terminal region 10. The RGD sequence in Npnt is critical for binding to its receptor, α8β1 integrin, and the interaction is critically involved in kidney development 10, 12. EGF‐like repeats and MAM domains are responsible for binding to chondroitin sulfate and heparan sulfate, respectively 13. The involvement of Npnt in diseases has been reported, particularly kidney diseases 14, 15, 16, 17, acute and chronic hepatitis 18, and tumor progression 19, 20, 21.

We performed real‐time PCR to evaluate Npnt expression in tissues and found that Npnt expression was high in the spleen. Therefore, we hypothesize that Npnt may play an important role in autoimmune diseases. To test this hypothesis, we developed an enzyme‐linked immunosorbent assay (ELISA) system for quantification of Npnt protein expression levels and generated an antibody against the α8β1 integrin‐binding site. In the present study, we show the expression and functional importance of Npnt in autoimmune diseases.

## Materials and methods

### Reagents and cell lines

Mouse nephronectin (Npnt) protein [called Npnt (R&D) protein in this study] was from R&D systems (Minneapolis, MN, USA). RD cells (derived from a human rhabdomyosarcoma), A549 cells (derived from human lung adenocarcinoma), HepG2 (derived from human hepatocellular carcinoma), LN‐229 cells (derived from human glioblastoma), B16 cells (derived from mouse skin melanoma), NIH3T3 cells, MEF cells, L929 cells (derived from mouse fibroblast) were cultured in DMEM containing 10% fetal bovine serum (FBS). Raji cells (derived from human Burkitt's lymphoma), Jurkat cells (derived from human T‐cell leukemia), U937 cells (derived from human histiocytic lymphoma), HL60 cells (derived from human acute promyelocytic leukemia), Ehrlich cells (derived from mouse Ehrlich‐Lettre ascites carcinoma), X63 cells (derived from mouse myeloma) were cultured in RPMI containing 10% FBS.

### Animals

Mice and rats were kept under specific pathogen‐free conditions and provided food and water *ad libitum*. Every effort was made to minimize suffering during injections, and all surgery was performed on humanely sacrificed animals. All animal experiments were performed in accordance with the guidelines of the Bioscience Committee of and were approved by the Animal Care and Use Committee of Immuno‐biological Laboratories.

Specific pathogen‐free BALB/c mice, C57BL/6, and SD rats were purchased from Japan SLC (Hamamatsu, Japan).

### Real‐time PCR

Total RNA from healthy mouse tissues, arthritic joints, and synovial cells was extracted with TRIzol (Thermo Fisher, Hanover Park, IL, USA). First‐strand cDNA was generated with a first‐strand cDNA synthesis kit (TOYOBO, Osaka, Japan). For human Npnt expression in human tissues, a human multiple tissue cDNA panel was used (Takara, Kusatsu, Japan). The specific primers used are shown in Table 1. The expression level of mRNA was calculated using the calibration curve method using lightcycler software version 3 (Roche Diagnostics, Indianapolis, IN, USA). Data were standardized to G3PDH.

**Table 1:** Real‐time PCR primer sequences.

Gene	Sequence of primers
Human Npnt	5′‐AAATGAGTGTGGCCTGAAGC‐3′
	5′‐TGCCTAAATCTAGGGCAGGA‐3′
Mouse Npnt	5′‐GCGGATGAGGAAGTAAAGGAC‐3′
	5′‐CCTTTGAAGATGACGCTTTTG‐3′
Human α8 integrin	5′‐TCTGCTGCACCCAATGATTA‐3′
	5′‐CTCCACAGTCCACCAGAATGT‐3′
Mouse α8 integrin	5′‐AGATTTGCTTGTAGGGGCATT‐3′
	5′‐GACAGCTTCAAGTCAGGAACG‐3′

### Npnt preparation

The human Npnt complementary DNAs (cDNAs) were PCR‐amplified from A549 cells. Primer sets for cloning of Npnt are shown in Table 2. The amplified product was cloned into a pM‐secSUMOstar vector (LifeSensors, Malvern, PA, USA), which contains a His‐tag and SUMO tag at the 5′ end of the cloning sites for purification and enhancing expression and solubility. The cDNAs encoding domain of human Npnt was subcloned into pM‐secSUMOstar in‐frame with a His‐tag and SUMO tag. EGF‐like repeats, Linker segment, and MAM domain were amplified by PCR using primer sets described in Table 2. The Npnt expression plasmids after nucleotide sequence confirmation were transfected into 293 cells with Lipofectamine 2000 (Thermo Fisher). His‐SUMO‐tagged human Npnt was purified using TALON Resin (Takara) following the manufacturer's protocol.

**Table 2:** Primer sequences for cloning of Npnt.

Gene	Sequence of primers
Human Npnt	5′‐TATTGAGGCTCATCGCGAACAGATTGGAGGTGAGTTCGACGGGAGGTGGCCC‐3′
	5′‐ATGCCTGCAGGTCGACTTAGCGTTCTTCAGAGCAGTG‐3′
EGF‐like repeats	5′‐TATTGAGGCTCATCGCGAACAGATTGGAGGTGAGTTCGACGGGAGGTGGCCC‐3′
	5′‐ATGCCTGCAGGTCGACTTACACACAAGTCAGTCCATCACC‐3′
Linker segment	5′‐TATTGAGGCTCATCGCGAACAGATTGGAGGTTATATCCCAAAAGTTATGATT‐3′
	5′‐ATGCCTGCAGGTCGACTTATACCAGAACACCTGGATCATC‐3′
MAM domain	5′‐TATTGAGGCTCATCGCGAACAGATTGGAGGTCACAGTTGTAATTTTGACCAT‐3′
	5′‐ATGCCTGCAGGTCGACTTAGCGTTCTTCAGAGCAGTG‐3′

### Production and purification of polyclonal and monoclonal antibodies against Npnt

Synthetic peptides used for immunization were as follows: Npnt3 peptide FKGEKRRGHTGEIGLDDVSL and NPNT‐FD peptide PQKPRGDVFIPRQPTNDLFEIFEIER, which correspond to common amino acid sequences in human and mouse Npnt, and the functional domain of mouse Npnt for α8β1 integrin, respectively. These peptides were used to immunize rabbits after coupling with thyroglobulin. The immunoglobulin (Ig) G fraction was obtained from a sepharose column coupled with the synthetic peptide used as the immunogen.

Generation of monoclonal antibodies against Npnt was as follows. SD rats were subcutaneously immunized with mouse Npnt protein mixed with Freund's complete adjuvant (Becton Dickinson, Sparks, MD, USA) (day 1) and immunized with mouse Npnt protein mixed with Freund's incomplete adjuvant (Becton Dickinson) (days 7, 14, and 21) Spleen cells were harvested and fused with X63‐Ag8‐653 cells in the presence of polyethylene glycol (Roche Diagnostics, Indianapolis, IN, USA). Hybridomas were screened for production of antibody specifically reactive to immobilized mouse Npnt used for the immunization, and one IgG1 clone (20D1a) from the mouse Npnt protein was obtained.

### Establishing the Npnt sandwich ELISA system

About 5 μg·mL^−1^ of rabbit anti‐Npnt3 antibody was used as a capture antibody, and 2 ng·mL^−1^ of 20D1 conjugated with HRP by using Peroxidase labeling kit according to the manufacturer's instructions (DOJINDO, Kumamoto, Japan) was used as the detection antibody. Purified mouse Npnt (R&D) protein was used as the standard. 100 μL of the diluted sample was applied to each well of the antibody‐coated plate and incubated for 1 h at 37 °C. The wells were washed three times with PBS‐T [PBS containing 0.05% (v/v) Tween‐20], and then, 100 μL of detection antibody was added and incubated for 30 min at 4 °C. Each well was washed with PBS‐T and incubated with 100 μL of 3,3′,5,5′‐tetramethyl benzidine (TMB) (Sigma‐Aldrich, St Louis, MO, USA) for 30 min, and then, 100 μL of 1 m sulfuric acid was added. Plates were read at a wavelength of 450 nm by an immunoreader (Thermo Fisher).

### Direct ELISA

Mouse Npnt (R&D), fibronectin (Sigma‐Aldrich, St Louis, MO, USA), GRGDS peptide‐coupled with BSA were coated onto 96‐well plate at a concentration of 1 μg·mL^−1^ at 37 °C for 1 h, then blocked with 0.1% BSA in PBS containing 0.05% NaN_3_ at 37 °C for at least 1 h. The plates were washed two times with PBS‐T and incubated with anti‐Npnt‐FD mAb at various concentrations at 37 °C for 1 h. After a further three washes, 1 : 5000 dilution of HRP‐conjugated anti‐mouse IgG antibody (Jackson Immuno Research, West Grove, PA, USA) was added to each well at room temperature for 30 min. Bound protein was quantified by a colorimetric assay using TMB as a substrate for 15 min at room temperature. Plates were read at a wavelength of 450 nm by an immunoreader.

### Cell adhesion assay

The 96‐well plates were precoated with hNpnt, mNpnt (R&D), NPNT‐FD synthetic peptides, fibronectin, or vitronectin (2 μg·mL^−1^) at 37 °C for 1 h, followed by treatment with 0.5% BSA in PBS for 1 h at room temperature. Cells in the presence or absence of various concentrations of antibody were suspended in DMEM containing 0.25% BSA, and 200 μL of cell suspension (at a cell density of 5 × 10^4^ cells/well) was applied to 96‐well plates and incubated for 1 h at 37 °C. The medium was removed from the plates, and all wells were washed twice. The adherent cells were fixed and stained with 0.5% crystal violet in 20% methanol for 30 min. All wells were rinsed three times with water, and adherent cells were then lysed with 20% acetic acid. The resulting supernatants from each well were analyzed by an immunoreader, and the absorbance at 595 nm was measured to determine the relative number of cells adhered to wells.

### Fractionation of synovial tissues

Hind limbs were surgically removed from the ankle joint, followed by surgical separation of the dermal, subcutaneous, tendinous, and muscle tissues from joints. The remaining soft tissues were removed from the bones and homogenized roughly with dissecting scissors. Homogenates were washed once with culture medium and treated with 3 mg·mL^−1^ type II collagenase (Worthington Biochemical, Freehold, NJ, USA) by vigorous stirring. After washing, synovial cells were fractionated into TLR‐4^+^ or TLR‐4^−^ fractions using MACS (Miltenyi Biotec, Auburn, CA, USA). The TLR‐4^+^ fraction was considered macrophages and the TLR‐4^−^ fraction was cultured overnight, and adherent cells were used as fibroblasts.

### Arthritis model

Arthritis was induced using an arthritogenic 5‐clone monoclonal antibody cocktail kit (Chondrex Inc., Redmond, WA, USA) following the manufacturer's protocol. Briefly, 7‐week‐old female BALB/c mice were injected intravenously with a mixture of five anti‐type II collagen monoclonal antibodies (2 mg each) on day −3, followed by an intraperitoneal injection of 50 µg of lipopolysaccharide (LPS) (0111:B4) on day 0. About 400 μg anti‐Npnt‐FD antibody or control rabbit IgG was administered intraperitoneally at doses of 400 μg per mouse on days −4 and 0. The clinical severity of arthritis was graded up to 9 days after LPS administration in each of the four paws on a 0–4 scale. The disease severity was recorded for each limb as follows: 0, normal; 1, focal slight swelling and/or redness in one digit; 2, moderate swelling and erythema of two digits; 3, marked swelling and erythema of the limb; and 4, maximal swelling, erythema, deformity, and/or ankylosis. Mice were scored in a double‐blind manner. Mice were sacrificed, and then, joint sections were stained with hematoxylin and eosin (HE) and fast green/safranin‐O for immunohistochemical evaluation.

### Induction of concanavalin A‐induced hepatitis and experimental autoimmune encephalomyelitis

For induction of ConA‐hepatitis, BALB/c mice were injected intravenously with 15 mg ConA (Sigma‐Aldrich) per kilogram of body weight, dissolved in pyrogen‐free PBS.

For induction of experimental autoimmune encephalomyelitis (EAE), C57BL/6 or SJL/J mice were injected subcutaneously with 100 µg of myelin oligodendrocyte glycoprotein (MOG) 35–55 peptide (MEVGWYRSPFSRVVHLYRNGK) or Myelin proteolipid protein (PLP) 139–151 (HSLGKWLGHPDKF) emulsified with complete Freund's adjuvant on day 0 and intravenous injection of 400 ng of pertussis toxin (List Biological Laboratories, Campbell, CA, USA) on days 0 and 2.

### Statistical analysis

Data are presented as means ± SEM and are representative of at least three independent experiments. The statistical significance of differences between groups was calculated with a two‐tailed Student's *t*‐test. Differences were considered significant for *P* < 0.05 (*) or 0.01 (**).

## Results

### Expression of Npnt and α8 integrin in normal tissues

**Figure 1:**
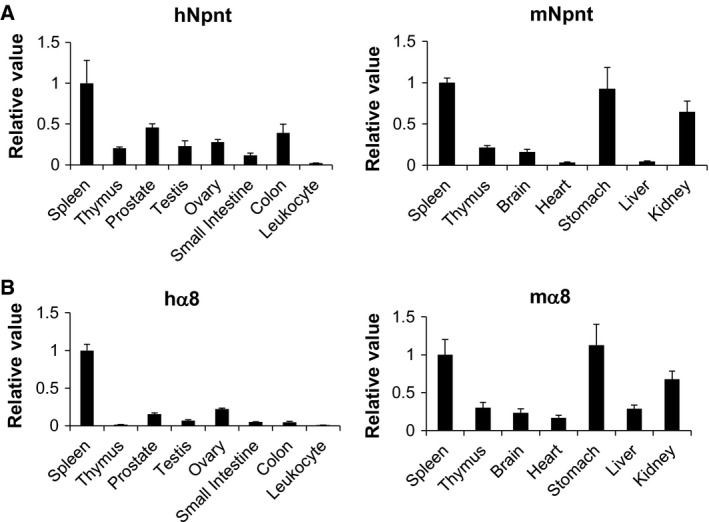
Expression of Npnt and α8 integrin in human and mouse tissues. Npnt (A) and α8 integrin (B) expression in healthy tissues were evaluated by real‐time PCR. Data are representative of three or more independent experiments with similar results. Data are presented as means ± SEM.

We first examined the expression level of human and mouse Npnt (Fig. 1A) and α8 integrin (Fig. 1B) in healthy human and mouse tissues using real‐time PCR. Subsequently, we found both Npnt and α8 integrin were highly expressed in the spleen; thus, we hypothesized that the interaction between Npnt and α8 integrin may be involved in immunological diseases.

### Establishing a sandwich ELISA system for Npnt

**Figure 2:**
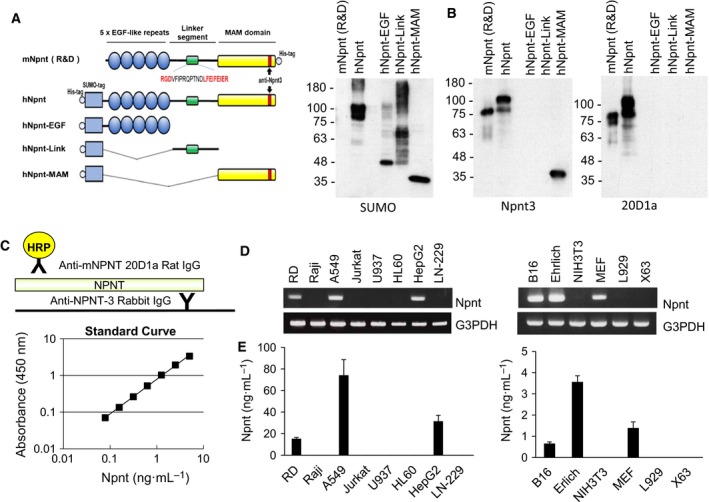
Establishing the sandwich ELISA system for Npnt. (A) Schematic representation of Npnt and its truncated variants. Cell binding sequences (RGD sequences) in the linker segment and the epitope in the MAM domain of anti‐Npnt3 antibody are indicated. (B) Specificity of Npnt3 and 20D1a antibodies by western blotting. (C) Diagram (top panel) and standard curve (lower panel) of the ELISA. Recombinant mNpnt (R&D) protein was used as the standard. (D) Expression of Npnt in cell lines by RT–PCR. Human or mouse cell lines were left or right panel, respectively. (E) Detection of Npnt in supernatants from cell lines used in (C). Data are representative of three or more independent experiments with similar results. Data are presented as means ± SEM.

To analyze the expression level of Npnt in an autoimmune mouse model, we sought to establish an Npnt sandwich ELISA system. For ELISA construction, two antibodies against Npnt were generated, Npnt3 rabbit polyclonal antibody and 20D1 monoclonal antibody. For characterization of these antibodies, we purified full length of Npnt, EGF‐like repeats, linker segment, MAM domain (Fig. 2A). Both antibodies were capable of detecting mouse and human Npnt (Fig. 2B). Notably, the molecular weight of human Npnt is higher than mouse because human Npnt is fused with a SUMO tag (approximate molecular weight of 20 kDa). A link truncated variant showed higher and broader bands than the expected size (approximately 28 kDa) as reported previously 22. Anti‐Npnt3 antibody recognized the MAM domain as predicted, whereas the 20D1 Ab did not react with a panel of Npnt truncated variants, suggesting that 20D1a Ab recognizes the Npnt conformation (Fig. 2B).

We developed an ELISA combination using Npnt3 as a coating antibody and 20D1 as a detecting antibody (Fig. 2C). The standard curve of the ELISA system exhibited a linear shape ranging from 0.078 to 5.0 ng·mL^−1^ calculated with mouse Npnt (R&D) protein (Fig. 2C). To analyze the specificity of the ELISA, Npnt mRNA expression in various human and mouse cell lines was explored by RT–PCR, and Npnt expression was detected in RD cells, A549 cells and HepG2 cells in human cell lines, and B16 cells, Ehrlich cells, and MEF cells in mouse cell lines (Fig. 2D). The ELISA we developed successfully detected secreted Npnt protein in the supernatant of the cell lines expressing Npnt by RT–PCR, but not from non‐Npnt mRNA expressing cell lines (Fig. 2E).

### Elevated expression of Npnt in autoimmune diseases

**Figure 3:**
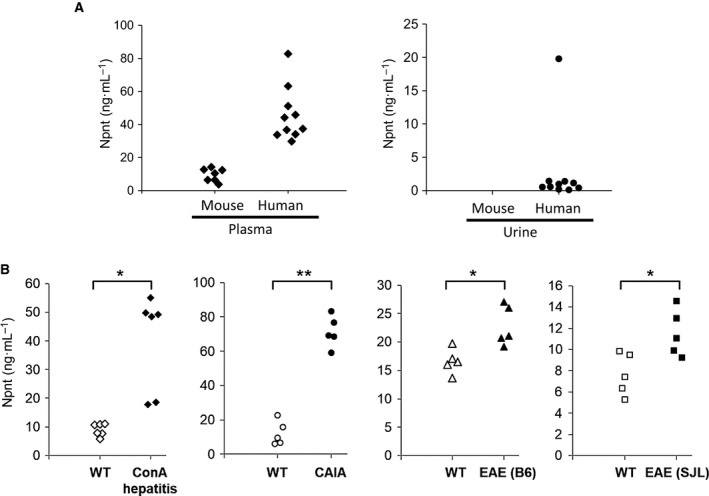
Npnt in plasma and urine samples. (A) Npnt in plasma and urine samples from healthy mice and humans. (B) Npnt in plasma samples from various murine autoimmune disease models. Plasma samples were tested on the day when inflammation was exacerbated as follows, for ConA‐hepatitis, 24 h after ConA injection; for CAIA, 7 days after LPS injection; for EAE, 14 days after MOG or PLP peptide immunization. Data are representative of three or more independent experiments with similar results. *P < 0.05 or **P < 0.01 versus plasma samples from wild‐type mice (Student's *t*‐test).

The antigenic peptide of anti‐Npnt3 antibody is a common sequence shared by humans, mice, and rats, and the 20D1a antibody recognizes both human and mouse Npnt. Therefore, we assayed Npnt values in healthy mouse, human, and rat plasma samples. We found that the Npnt system can be used for quantification of mouse and human Npnt values with Npnt (R&D) protein as the standard. We next evaluated Npnt expression in urine since Npnt is highly expressed in kidneys. Significant expression of Npnt in human urine, but not mouse urine, was detected (Fig. 3A). Next, we tested Npnt levels in plasma samples from autoimmune mouse models, specifically ConA‐hepatitis, CAIA, and EAE models. We found that Npnt levels in all of the inflammatory models we tested were significantly elevated (Fig. 3B).

### Involvement of α8β1 integrin‐binding region of Npnt in inflammatory arthritis

**Figure 4:**
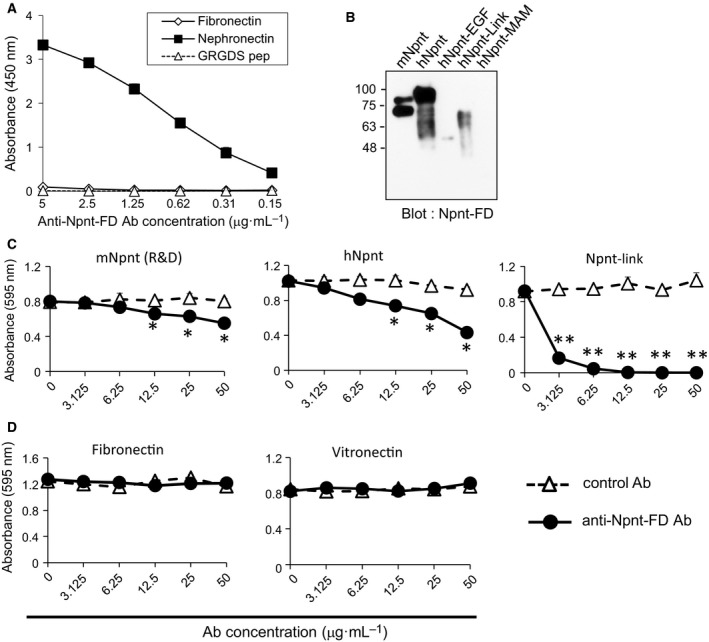
Generation of neutralizing antibodies for Npnt. (A) Cross‐reactivity of anti‐Npnt‐FD antibody to plates coated with Npnt, fibronectin, or GRGDS peptide‐coupled with BSA (1 μg·mL^−1^) by direct ELISA. (B) Specificity of anti‐Npnt‐FD antibody by western blotting. (C, D) NIH3T3 cells were allowed to adhere to 96‐well plates precoated with full‐length of Npnt or Npnt‐Link (C), fibronectin, or vitronectin (D) in the presence of indicated concentrations of anti‐Npnt‐FD antibody. Normal rabbit IgG was used as a control antibody. Data are presented as mean ± SEM. Data are representative of three or more independent experiments with similar results. *P < 0.05, anti‐Npnt‐FD antibody versus control antibody (Student's *t*‐test).

The RGD motif and LFEIFEIER sequence in Npnt are required for binding to α8β1 integrin, which is the physiological receptor for Npnt. To assess the importance of the α8β1 integrin‐binding region within murine Npnt, we prepared the anti‐Npnt‐FD antibody that recognizes the PQKPRGDVFIPRQPTNDLFEIFEIER motif used as the antigen. We first examined the specificity of the anti‐Npnt‐FD antibody by direct ELISA, western blotting, or cell adhesion test. We also asked whether Npnt‐FD antibody cross‐reacts with fibronectin or GRGDS peptide since RGD motif found in many ECM molecules is exist in antigen peptide. The anti‐Npnt‐FD antibody recognized mouse Npnt protein, but not RGD motif‐containing ECM, fibronectin or GRGDS peptide by direct ELISA (Fig. 4A), and detected mouse Npnt, human Npnt, and the Linker segment, which contains the antigen region of the anti‐Npnt‐FD antibody, by western blotting (Fig. 4B). To examine the functional specificity, cell adhesion assays were performed. Anti‐Npnt‐FD antibody partially inhibited cellular adhesion to mouse and human Npnt, whereas it completely inhibited the adhesion of the α8β1 integrin‐binding region, suggesting that an alternative Npnt exists (Fig. 4C). Anti‐Npnt‐FD antibody did not show any inhibitory activity toward fibronectin or vitronectin (Fig. 4D). These results indicate that anti‐Npnt‐FD antibody specifically recognizes and functionally inhibits the interaction of the α8β1 integrin‐binding region of Npnt with its receptor.

**Figure 5:**
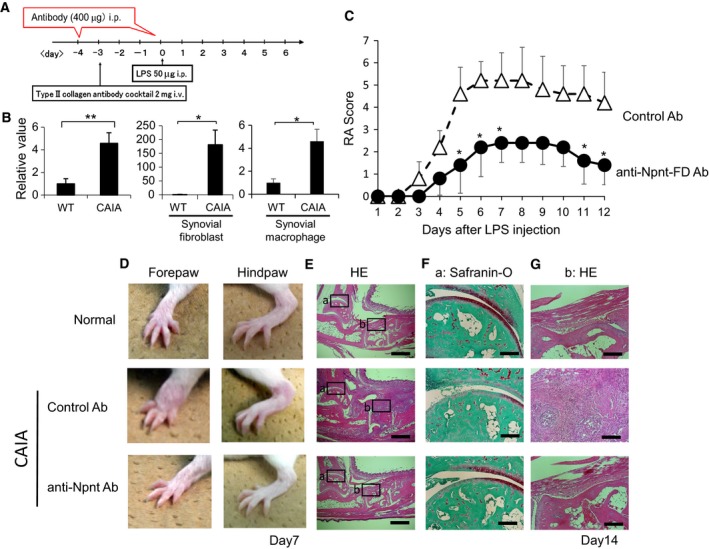
Npnt neutralization by anti‐Npnt‐FD antibody ameliorates CAIA. (A) Protocol for CAIA with anti‐Npnt‐FD antibody treatment. The antibody was administered to BALB/c mice on days −4 and 0 relative to LPS injection during CAIA induction. (B) Npnt expression in arthritic joints on day 6, synovial fibroblasts, or synovial macrophages were evaluated by real‐time PCR. *P < 0.05, **P < 0.01, wild‐type versus CAIA (Student's *t*‐test). (C) Arthritis scores of arthritic mice treated with control antibody (rabbit IgG) or an anti‐Npnt‐FD antibody at the indicated time points (*n* = 5 per group). *P < 0.05, **P < 0.01, anti‐Npnt‐FD antibody versus control Ig (Student's *t*‐test). Data are presented as means ± SEM. (D) Representative images of the forepaw and hindpaw on day 7 are shown. (E–G) Representative histology of normal joints and arthritic joints on day 14 from mice treated with control antibody or anti‐Npnt‐FD antibody. Sections were stained with HE (E, G) or Safranin‐O (F). Magnified views of the boxed areas a and b are shown in F and G, respectively. Scale bar = 2mm (E), 200μm (F and G). Data are representative of three or more independent experiments with similar results.

An antibody against OPN, which is one of the α8β1 integrin ligands, inhibits an exacerbation of CAIA; thus, we assessed the function of the anti‐Npnt‐FD antibody in the CAIA model 23. In the CAIA model, on day 6 the Npnt mRNA in arthritic joints was elevated. Furthermore, synovial fibroblasts and synovial macrophages, which are the main cellular components in arthritic synovia and important players in the joint pathology of rheumatoid arthritis (RA) 24, 25, were elevated (Fig. 5B). Treatment with the anti‐Npnt‐FD antibody on days −3 and 0 led to the delay of clinical onset of arthritis, and the severity of arthritis was significantly attenuated (Figure 5C). The gross appearance of arthritic joint treated with anti‐Npnt‐FD antibody on day 7 is shown in Fig. 5D. Finally, we evaluated the effect of anti‐Npnt‐FD antibody treatment on joint histology on day 14 after the LPS injection. In concert with the clinical score, histological analysis demonstrated marked differences. Cartilage destruction evaluated by Safranin‐O staining was faint in the arthritic joints of control mice. In contrast, the staining was partially rescued in anti‐Npnt‐FD antibody‐treated mice (Fig. 5F), indicating that the destruction of proteoglycans was partially prevented by the anti‐Npnt‐FD antibody. The proliferation of synovial cells, evaluated by HE staining, was detected in control antibody‐treated mice; however, this feature of CAIA was clearly diminished following the administration of the anti‐Npnt‐FD antibody (Fig. 5G). These results strongly suggested that the α8β1 integrin‐binding region of Npnt is involved in inflammatory arthritis.

## Discussion

In the present study, we investigated the function of Npnt in autoimmune diseases as high levels of Npnt mRNA were found in the spleens of humans and mice. We developed a sandwich ELISA system for quantifying the protein level of Npnt in autoimmune diseases and found that Npnt in blood is increased in various autoimmune diseases. The results from ConA‐hepatitis are consistent with previous reports that observed elevated Npnt mRNA 18.

For clarification of Npnt function in autoimmune diseases, we generated a neutralizing antibody against Npnt (anti‐Npnt‐FD antibody) by immunizing rabbits with the PQKPRGDVFIPRQPTNDLFEIFEIER peptide (Npnt‐FD peptide), because the RGD motif and LFEIFEIER sequence in Npnt plays a critical role in the binding to its physiological receptor, α8β1 integrin 22. Anti‐Npnt‐FD antibody partially inhibits cell adhesion of full‐length mouse and human Npnt to NIT3T3 cells, which express α8β1 integrin 26. This suggests that Npnt has other receptors on NIH3T3 cells. In fact, it has been reported that the MAM domain in Npnt binds to heparan sulfate proteoglycan and EGF‐like repeats domain and activates the EGF receptor 13, 27. Therefore, anti‐Npnt‐FD antibody specifically inhibits the interaction between Npnt and receptors bound to Npnt‐FD peptide such as α8β1 integrin.

Previously, we reported that neutralizing antibodies for osteopontin, one of the ligands of α8β1 integrin, inhibits the development of inflammatory arthritis in the CAIA model. Therefore, we analyzed the function of Npnt in CAIA and determined that anti‐Npnt‐FD antibody attenuates the severity of arthritis. We also found higher expression of Npnt in arthritic joints than in control mice. In RA joints, proliferating fibroblasts and macrophages are clearly seen. These cells are thought to cause an exacerbation of RA by secretion of various pro‐inflammatory cytokines such as tumor necrosis factor (TNF)‐α or interleukin (IL)‐1β. In this study, Npnt expression in synovial fibroblasts and macrophages from CAIA was also increased, suggesting a critical involvement of the PQKPRGDVFIPRQPTNDLFEIFEIER sequence of Npnt and its receptor(s) in the development of inflammatory arthritis and, perhaps, the combination of Npnt and these cytokines have a synergistic effect on the severity of inflammation.

The α8β1 integrin, which is part of the RGD‐recognizing integrin family, is prominently expressed in smooth muscle and has a defined in vivo role in kidney development and liver and lung fibrosis 10, 11, 28, 29. In this study, α8β1 integrin is highly expressed in human and mouse spleen, suggesting that α8β1 integrin has any important role in immune responses. It is conceivable that the binding of Npnt to α8β1 integrin is involved in α8β1 integrin, but we cannot exclude the possibility that other RGD‐recognizing integrins, such as αvβ3 integrin, are the Npnt receptor during inflammatory arthritis. Further research is required to understand how α8β1 integrin is involved in inflammatory arthritis.

In conclusion, we found that Npnt is highly expressed in the spleen and upregulated in blood from various autoimmune mouse models. Furthermore, we provide evidence for a potential role of the α8β1 integrin‐binding region of Npnt in the pathogenesis of inflammatory arthritis. Further studies will be directed at understanding the mechanism by which Npnt and its interactions with integrins affect autoimmune diseases.

## Conflict of interest

SK and MH declare no conflicts of interest associated with this manuscript. KI, MM, and TS are employees of the Immuno‐Biological Laboratories Co., Ltd.

## Author contributions

SK conceived and carried out experiments and wrote the manuscript; MH performed experiments; KI, MH, MM, and TS generated anti‐Npnt antibody and established the Npnt ELISA system; SK and TS supervised the study. All authors read and approved the final manuscript.
